# Riluzole neuroprotection in a parkinson's disease model involves suppression of reactive astrocytosis but not GLT-1 regulation

**DOI:** 10.1186/1471-2202-13-38

**Published:** 2012-04-05

**Authors:** Marica Carbone, Susan Duty, Marcus Rattray

**Affiliations:** 1 King's College London, Wolfson Centre for Age-Related Diseases, Guy's Campus, London SE1 1UL, UK; 2 Reading School of Pharmacy, University of Reading, Whiteknights, Reading RG6 6UB, UK

**Keywords:** EAAT2, GLT-1, Neuroprotection, Parkinson's Disease, GFAP, Glial cell, 6-hydroxydopamine

## Abstract

**Background:**

Riluzole is a neuroprotective drug used in the treatment of motor neurone disease. Recent evidence suggests that riluzole can up-regulate the expression and activity of the astrocyte glutamate transporter, GLT-1. Given that regulation of glutamate transport is predicted to be neuroprotective in Parkinson's disease, we tested the effect of riluzole in parkinsonian rats which had received a unilateral 6-hydroxydopamine injection into the median forebrain bundle.

**Results:**

Rats were treated with intraperitoneal riluzole (4 mg/kg or 8 mg/kg), 1 hour before the lesion then once daily for seven days. Riluzole produced a modest but significant attenuation of dopamine neurone degeneration, assessed by suppression of amphetamine-induced rotations, preservation of tyrosine hydroxylase positive neuronal cell bodies in the substantia nigra pars compacta and attenuation of striatal tyrosine hydroxylase protein loss. Seven days after 6-hydroxydopamine lesion, reactive astrocytosis was observed in the striatum, as determined by increases in expression of glial fibrillary acidic protein, however the glutamate transporter, GLT-1, which is also expressed in astrocytes was not regulated by the lesion.

**Conclusions:**

The results confirm that riluzole is a neuroprotective agent in a rodent model of parkinson's disease. Riluzole administration did not regulate GLT-1 levels but significantly reduced GFAP levels, in the lesioned striatum. Riluzole suppression of reactive astrocytosis is an intriguing finding which might contribute to the neuroprotective effects of this drug.

## Background

The primary pathological event in Parkinson's disease is degeneration of the nigrostriatal dopamine neurons. In Parkinson's disease, corticostriatal and subthalamonigral glutamate systems are hyperactive, and contribute to symptoms and dopamine neuronal death, through the process of "excitotoxicity" [1-3]. Reducing glutamate transmission through activating type III metabotropic glutamate receptors [2,4], blocking type I metabotropic glutamate receptors [5], or blocking postsynaptic AMPA or NMDA receptors [6,7] have been shown to be neuroprotective and reduce motor symptoms in animal models of Parkinson's disease. In general, therefore, anti-glutamate approaches are attractive and promising therapeutically, although they have not yet led to effective clinical drugs.

One potential way of regulating glutamate levels, which has been largely overlooked in Parkinson's disease, is by modulating astrocytes. Astrocytes express glutamate transporters which remove glutamate from the synaptic cleft and thus control the duration and magnitude of glutamate's actions [8]. In Parkinson's disease astrocytes show features typical of reactive astrocytosis [9]. In animal models of Parkinson's disease, astrocytes are also markedly altered: following dopamine neurone degeneration astrocytes become reactive, as seen by a sustained and progressive increase in the levels of the astrocyte cytoskeletal protein, glial fibrillary acidic protein (GFAP) together with changes in astrocyte morphology [10-12]. Since reactive astrocytosis is associated with loss of the protective functions in astrocytes [13], suppression of astrocyte reactivity may be beneficial in disease.

We recently identified the clinically used neuroprotective compound, riluzole, as a positive modulator of the major astrocyte glutamate transporter, GLT-1, *in vitro *[14]. Riluzole has already been through a number of clinical trials in Parkinson's disease patients [15-17]. The largest clinical study published to date was a double-blind study with 20 patients with early Parkinson's disease, untreated with L-dopa who received 100 mg riluzole per day for up to one year [17]. Here riluzole did not produce any significant improvement in disability or reduce the time before patients commenced dopamine agonist therapy, however the effects of riluzole on disease progression were not assessed. We therefore tested riluzole in the 6-hydroxydopamine (6-OHDA) treated rat model of Parkinson's disease to determine whether it can cause neuroprotection and whether protection was associated with modulation of GLT-1 expression in astrocytes.

## Results

**Figure 1:**
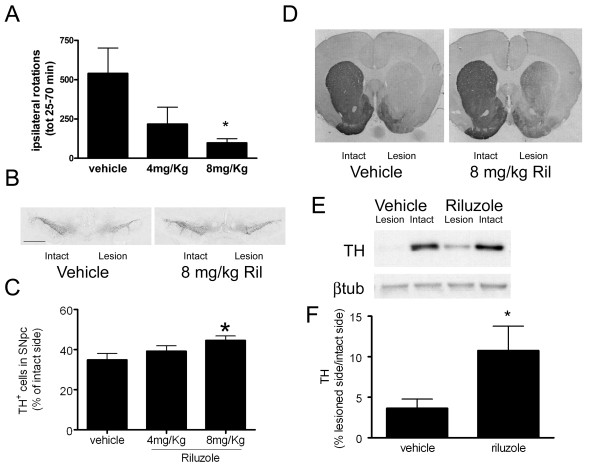
**Neuroprotective effects of riluzole (4 or 8 mg/kg) given 1 hour before and for 7 days post-lesion in the 6-OHDA rat model of PD**. A. Effect of riluzole or vehicle on ipsilateral rotations in lesioned rats measured between 25-70 minutes after amphetamine administration. B. Tyrosine Hydroxylase (TH) immunoreactive neurones in the substantia nigra pars compacta (SNpc), scale bar = 0.5 mm. C shows quantification of TH positive cells in SNpc on the side ipsilateral to the lesion ± SEM, expressed as a % of the unlesioned, intact side (n = 8 per group), * = p < 0.05, One-Way Anova followed by Bonferroni's test. D. Sections at the level of the striatum stained for TH immunoreactivity. E. Western blot data shows loss of TH immunoreactivity on lesioned side which is reduced in animals treated with riluzole (8 mg/kg). F shows these data quantified: for each animal the TH band intensity is divided by the band density of a housekeeping protein, β-tubulin and expressed as % of the intact side ± SEM (n = 8), * = p < 0.05, unpaired t-test.

Due to the functional deficit of dopamine transmission on the ipsilateral, lesion hemisphere following unilateral 6-OHDA lesion, animals show a high level of ipsiversive rotation when challenged with amphetamine (Figure 1A). Riluzole produced a significant, dose-dependent reduction in this effect, indicating functional preservation of the dopaminergic system. In support of this behavioural evidence, immunostaining revealed that riluzole (8 mg/kg, but not 4 mg/kg) significantly protected TH positive cells in the substantia nigra pars compacta from 6-OHDA induced cell death (Figure 1B, C). In vehicle treated animals, there were an average of 81 ± 6.6 cells (n = 8) counted on the unlesioned side, compared to 27 ± 2.1 cells on the lesioned side (n = 9). Riluzole (8 mg/kg) reduced the 6-OHDA-induced cell loss on the ipsilateral side from 66.1 ± 2.6% (n = 8) to 55.8 ± 4.5% (n = 8) (p < 0.05). Analysis of Western blots showed that riluzole (8 mg/kg) was able to partially preserve striatal TH levels from lesion-induced depletion (Figure 1E-F): after riluzole administration, the amount of TH remaining on the side ipsilateral to the lesion was approximately 4 times higher than when vehicle was administered.

**Figure 2:**
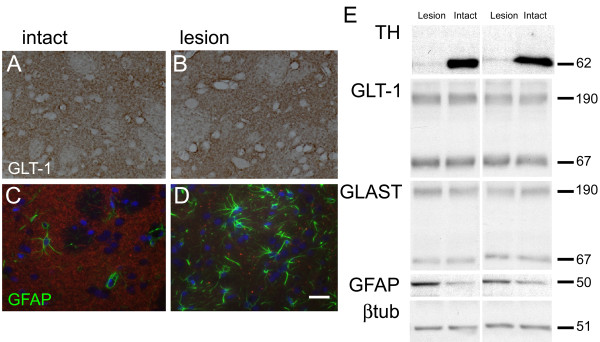
**Effect of 6-OHDA on neurochemical markers in striatum**. Panels A-D are photomicrographs of striatum seven days following 6-OHDA administration, showing GLT-1 immunostaining in striatum on lesioned (A) and intact (B) side and GFAP immunostaining - green on lesioned (C) and intact (D) side. Scale bar = 50 μm. E shows Western blots of striatal protein samples taken from 6-OHDA lesioned animals. Blots from two animals are shown for comparison. Membranes were blotted with antibodies against tyrosine hydroxylase (TH), GLT-1, GLAST, GFAP or beta-tubulin (βtub). Molecular weights (KDa) of protein bands are indicated, the glutamate transporters GLT-1 and GLAST are found as monomeric (67 KDa) and multimeric (ca. 190 KDa) species.

We next examined the astrocyte response to unilateral 6-OHDA lesion at 8 days after lesion, and the effect of riluzole administration. Immunostaining of sections suggested that 6-OHDA lesion caused no change in the expression of the glutamate transporter, GLT-1 in the striatum on the lesioned side (Figure 2A, B), although it caused a prominent increase in reactive astrocytes, as determined by GFAP immunostaining (Figure 2C, D). Western blotting confirmed these observations: there was no change in the striatal levels of GLT-1 protein following lesions that produced complete loss of tyrosine hydroxylase, and upregulated striatal GFAP (Figure 2E).

**Figure 3:**
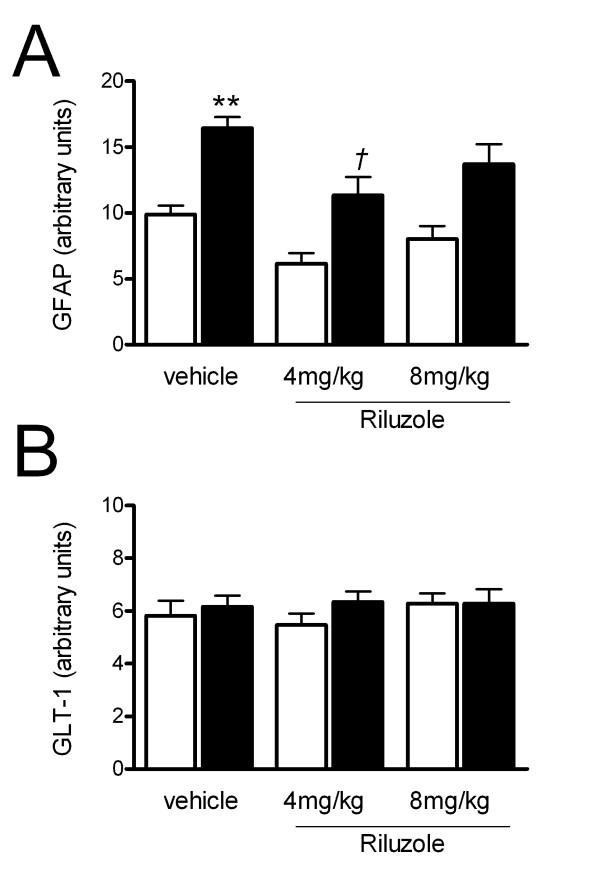
**Effect of riluzole on astrocyte protein expression in the striata of animals bearing a 6-OHDA lesion**. Western blots were quantified, and band density of GFAP and GLT-1 calculated relative to the housekeeping protein, β-tubulin. Graphs show the relative expression levels of each of these proteins on the intact side (open bars) and lesioned side (filled bars) in 6-OHDA lesioned animals ± SEM (n = 8-9) ** = p < 0.01 compared to vehicle-treated intact side, *† *= p < 0.05 compared to vehicle-treated lesioned side.

Figure 3 shows the quantification of the effect of lesion and/or riluzole treatment on GFAP and GLT-1. As shown in Figure 3A, 6-OHDA lesion caused an increase in GFAP protein on the striatum on the lesioned side to 166 ± 8% (n = 8, p < 0.01) compared to the intact side (Figure 3A). In animals treated with riluzole (4 or 8 mg/kg/day), there was still a significant increase in GFAP levels on the lesioned side compared to the intact side, however the increase in striatal GFAP on the side of the lesion was significantly reduced in animals treated with 4 mg/kg/day riluzole compared to vehicle treated animals by 31 ± 9% (n = 9, p < 0.05). Treatment of lesioned animals with 8 mg/kg/day riluzole also suppressed lesion-induced elevation of GFAP by 17 ± 9%, though this increase was not statistically significant. In these animals, GLT-1 levels were unaffected by lesion and/or riluzole treatment (Figure 3B). GLAST levels were unaffected by lesion or by treatment with riluzole (data not shown).

## Discussion

We tested riluzole for its ability to confer neuroprotection in the 6-hydroxydopamine rat, an animal model of Parkinson's disease. Our finding, that riluzole protects dopamine neurones from cell death confirms and extends the data reported in rats with a unilateral 6-OHDA (6 μg) lesion which showed that a single riluzole injection (8 mg/kg) before lesion, and one twenty-four hours afterwards suppressed apomorphine or amphetamine-induced rotations and significantly reduced the loss of dopamine in the striatum [18]. Riluzole has also consistently been demonstrated to provide neuroprotection in MPTP-treated mice, when administered before the lesion [19-21]. In non-human primates riluzole administration either 1 hour prior to, or one hour after MPTP administration, followed by additional administrations also caused neuroprotection [22-25]. Our data therefore confirms that riluzole is an effective neuroprotective agent, at least when administration commences 1 h before the experimental lesion.

Motor symptoms of Parkinson's Disease appear when around 60-70% of the nigrostriatal tract has degenerated, in this study a partial lesion was used to mimic early stage Parkinson's Disease, when neuroprotective strategies might be employed in the clinic. Such lesions have been successfully adopted in other studies of this kind in our lab [26]. Nevertheless, we acknowledge that there are limitations to the animal model used, not least because the protective drug is administered before lesioning. This pre-lesion administration is not clinically relevant but helps identify whether particular drug targets and mechanisms are likely to be effective if appropriate timing of administration can be achieved with better pre-symptomatic diagnosis, and is a protocol employed in many studies. The currently available animal models are an acknowledged limitation in the field [27]. The rapidity of cell death which occurs following experimental administration of dopamine neurotoxins means that the true mechanisms underlying dopamine cell death are still being debated.

The current study reveals a significant, but modest, neuroprotection when measured by protection of tyrosine hydroxylase positive cells, yet a larger reduction in behavioural rotations. As recently reviewed [27], behavioural responses, including amphetamine-induced rotation readouts obtained from animals bearing a partial lesion of the type used here can be rather variable. Thus, although amphetamine induces ipsiversive rotations, this may be seen in as few as half the animals tested [28], and the degree of rotation does not always change in direct relation to the degree of cell loss across a wide range of between 50-90% [29-31]. In our study, protection from 66 to 56% cell loss is reflected in a significant reduction in amphetamine-induced rotations, making it a useful, but not sole indicator of the degree of neuroprotection achieved. However, this result signifies that even a modest degree of cell protection, as achieved here, can manifest as functional improvement in motor behaviour if the resultant level of cell preservation moves beyond the threshold for symptom generation.

While efficacious in rodent and primate models, riluzole has not proved clinically useful in patients with Parkinson's disease [17]. The apparent discrepancy between the data sets most likely relates to the doses used. While it is difficult to accurately scale doses from humans to lower species, the riluzole doses used in this study (4 mg/kg or 8 mg/kg), typical of the doses required to produce positive effects in rodents [25,32], are five to ten fold higher than the dose of riluzole used clinically for the treatment of Amyotrophic Lateral Sclerosis/Motor Neuron Disease (ALS/MND). In ALS/MND patients, 50 mg twice daily is administered, i.e. between 1-2 mg/kg. This dose has been evaluated as providing the best benefit:risk ratio in ALS/MND [33], though doses of up to 200 mg/day are tolerated [33,34]. Riluzole doses such as those used clinically have never been reported to be effective in primate models of Parkinson's disease, indeed the primate data suggests that higher doses of riluzole, between 4-10 mg/kg daily are required for efficacy [22-25], i.e. equivalent to a dose range in man between 200 mg/day and 1 g/day. A 100 mg/day dose was shown to be ineffective in a clinical trial in patients with Parkinson's disease [17], no dose-ranging was carried out, and it is unknown whether higher doses would provide benefit. It is possible, therefore, that in patients an efficacious riluzole concentration may not have been achieved, through concerns about the adverse effects of higher doses. Though riluzole itself, because of its tolerability in man, may never be effective in Parkinson's disease, the proof of concept obtained in rodent and primate studies, including this one suggests that the drug is targeting a mechanism which is important in disease progression, thus suggesting routes for future therapies.

Riluzole's neuroprotective effects are generally regarded to be caused by its effects on reducing glutamate release in neurones following inhibition of voltage-gated sodium cation channels [35]. However, sodium channel blockers such as lamotrigine are ineffective in animal models of PD [36]. In addition, riluzole has additional, incompletely-characterised pharmacological effects which may be independent of effects on the persistent sodium current e.g. [37]. Since we had demonstrated that riluzole increases levels and activity of the glutamate transporter GLT-1 in primary cultures of striatal astrocytes [14], we reasoned that this transporter may be regulated by riluzole in vivo. However this study clearly demonstrates that riluzole does not regulate GLT-1 within one week of this partial 6-OHDA lesion.

Our data shows that, even though striatal astrocytes become reactive following 6-OHDA lesion there is no regulation of GLT-1 protein levels. This observation is unexpected and contrasts to the loss of GLT-1 found in reactive astrocytes *in vivo *in other animal models of neurodegeneration, for example a transgenic model of motor neurone disease [38]. The underlying mechanisms which determine extent of loss of GLT-1 in reactive astrocytes are incompletely understood, but are likely to relate to the extent of astrocyte activation and the degree of preservation of the afferent input to astrocytes, which support continued GLT-1 expression [39]. In the current study, we find GLT-1 to be neither up- nor down- regulated following 6-OHDA lesion, here measured 7 days after initial lesion. This is similar to the observations by others that there is no change in GLT-1 mRNA following striatal 6-OHDA lesion of rats [40] or in MPTP-treated mice [41]. However, there is some controversy on this issue; a recent well-controlled study shows that GLT-1 is upregulated after unilateral 6-OHDA lesions (16 μg) in rats 3 weeks and 12 weeks, but not 5 weeks after lesion [42], and the changes when present were found bilaterally, i.e. on the lesioned and unlesioned side, even though GFAP was significantly increased only in the striatum on the lesioned side. There are other studies which suggest that glutamate uptake activity [43] and GLT-1 protein is down-regulated after more substantial 6-OHDA lesions [44], or after MPTP treatment of mice [10]. We cannot resolve these apparent discrepancies at this time, though note there are differences in the lesion protocols: in this study we chose a partial lesion that resulted in around 60% depletion of striatal TH, nor did we analyse the striatum at later time points, when later changes may potentially occur. We note that, in the animals used in this study, the degree of activation of astrocytes is likely to be relatively mild compared to studies with a more complete dopamine neurone lesion.

Following 6-OHDA lesion there was a significant elevation of the astrocyte marker, GFAP, as expected [10-12]. Increased GFAP is usually interpreted as a marker of reactive astrogliosis, a phenotypic changes which include increased GFAP expression and elaboration of astrocyte processes [45]. Here we show quantitatively that riluzole has a marked effect on astrocytes in a model of neurodegeneration, as shown by its ability to reduce GFAP levels, both in the intact and lesioned striatum. Other groups have also suggested that riluzole treatment alters astrocyte morphology and GFAP expression in MPTP-treated mice [20,46]. It is possible that the ability of riluzole to suppress reactive astrocytosis may contribute to the neuroprotective effects observed in this study. As well as glutamate uptake, astrocytes exert a number of protective effects on neurones, and riluzole-induced reduction in astrocyte reactivity may lead to a suppression of neuroinflammatory pathways, a reduction in oxidative stress, hypotheses which can be tested in future studies.

## Methods

Male Sprague Dawley rats (B & K or Harlan, U.K.) weighing 270 - 300 g were used in these studies. Food and water were provided *ad libitum*. Animals were housed in a temperature- and humidity-controlled environment with a 12-h light/dark cycle. All procedures conformed to the U.K. Animals (Scientific Procedures) Act, 1986 and institutional ethical approval. For unilateral lesions, rats were pre-treated with desipramine (25 mg/kg i.p.) and pargyline (5 mg/kg i.p.) 30 minutes prior to induction of isoflurane anaesthesia. In fully anesthetised animals, 8 μg 6-OHDA (dissolved in 2.5 μl of 0.02% ascorbic acid/0.9% saline) was infused into the right median forebrain bundle (MFB) at a rate of 0.5 μl/min via an infusion pump. The stereotaxic coordinates used were: AP: -2.8, ML: +2.0, DV: -9.0 (from skull surface) relative to bregma with the incisor bar set at -3.3 mm [4]. Animals were treated with riluzole (4 or 8 mg/kg/kg, i.p., Tocris, Avonmouth UK) or PBS vehicle one hour prior to the first 6-OHDA injection and daily for 7 days thereafter.

We chose our lowest dose on the basis of previous work in rodent stroke models which used 4 mg/kg to see protective effects [32], and the highest dose on the basis of doses which have already been suggested to show protection in PD models, for example a recent primate studies which used 10 mg/kg riluzole to produce protective effects [25].

The extent of motor impairment in animals was assessed using amphetamine-induced rotational behaviour testing at 7 days post-lesion. Animals were harnessed in jackets tethered to an automated rotometer, placed in 40 cm diameter bowls and recorded for a 10 min baseline period before injection of d-amphetamine (5 mg/kg i.p.). Full 360° ipsiversive rotations were recorded in 5-min intervals for up to 90 min post-injection for subsequent assessment by observers blinded to treatment.

For immunostaining, on the final day of dosing (day 8), animals were terminally anaesthetised using pentobarbital (100 mg/kg) then trans-cardially perfused with 0.1 M PBS, followed by 4% paraformaldehyde in 0.1 M PBS. The brains were removed and stored in PFA at 4°C. Coronal sections (15 μm) were cut on a vibrating microtome and free floating sections were incubated with blocking buffer (1% normal goat serum in 0.1 M PBS) then overnight with rabbit anti -tyrosine hydroxylase (TH) antibody (1:1250; Chemicon), rabbit anti-glial fibrillary acidic protein (GFAP) antibody (1:1000, Dako), rabbit anti-GLT-1 (1:4000, gift of D. Pow, Brisbane). After three PBS washes, sections were incubated for 1 h with secondary antibody (biotinylated goat anti-rabbit 1:200; Sigma) then incubated for 30 min with an ABC kit (Vector Labs) and the signal developed in 10% diaminobenzidine tetrahydrochloride (DAB) in Tris-buffered saline for 10 min. In some experiments, the fluorescent secondary antibody, anti-rabbit Alexa 488 (1:1000) (Invitrogen, Paisley, UK) was used. Immunostained sections were viewed on a Zeiss apotome microscope and recorded using Axiovision LE software (Carl Zeiss Ltd., Hertfordshire, UK) at 50× magnification. To obtain cell counts in the substantia nigra pars compacta (SNpc), only viable TH-positive cells (i.e. intact round cells displaying a clear nucleus and cytoplasm) were counted in each hemisphere using image analysis software (Image J, NIH, Bethesda, MD). Previous studies have shown that data obtained using full stereological counting and manual counting of TH-positive cells in the SNpc in the 6-OHDA rat model are indistinguishable [47], so manual counting was adopted here. For each animal, the numbers of cells in three adjacent sections of SNpc was counted in both ipsilateral and contralateral hemispheres at three different rostrocaudal level (-4.8, -5.3, and -5.8 mm AP from Bregma), and averaged to give a mean cell count per section for the ipsilateral and contralateral sides. TH-positive cell counts in the 6-OHDA lesioned side, expressed as % of the contralateral, intact hemisphere, were compared between treatment groups using a One-way ANOVA and Bonferroni post-hoc test.

For Western blotting, on the final day of dosing rats were killed by terminal anaesthesia, and the brains rapidly dissected and regions frozen on dry ice. Western blotting of samples was carried out as described elsewhere [48]. Membranes were incubated with the following antibodies: rabbit anti GFAP (1:1000, Dako), rabbit anti-GLT-1 (1:4000, gift of D. Pow, Brisbane) or mouse anti-beta tubulin (1:400, Sigma-Aldrich). Detection was carried out using a horseradish peroxidase-conjugated goat anti-rabbit or anti-mouse IgG, as appropriate (1:1000, Sigma) using ECL Western blotting detection reagents and film detection (GE Healthcare). Bands were analysed using ImageJ (NIH, Bethesda, MD). GLT-1 quantification was obtained by quantifying both the lower (monomeric) and the high molecular mass (multimeric) bands, as described previously [49].

## Abbreviations

6-OHDA: 6-hydroxydopamine; AMPA: 2-amino-3-(5-methyl-3-oxo-1,2- oxazol-4-yl)propanoic acid; ANOVA: Analysis of Variance; EAAT2: Excitatory amino acid transporter 2; GFAP: glial fibrillary acidic protein; GLT-1: glutamate transporter-1 (EAAT2); MPTP: 1-methyl-4-phenyl-1,2,3,6-tetrahydropyridine; NMDA: N-methyl-D-aspartic acid; PD: Parkinson's Disease; TH: tyrosine hydroxylase.

## Competing interests

The authors declare that they have no competing interests.

## Authors' contributions

MC participated in the study design, carried out all of the experiments described in the study, was involved in the analysis and interpretation of the data and production of the figures. SD participated in the study design and supervised the in vivo arm of the study, was involved in the analysis and interpretation of the data and writing the manuscript. MR participated in the study design, analysis and interpretation of the data, produced the figures and drafted the manuscript. All authors read and approved the manuscript.

## Authors' information

MC was a postdoctoral research fellow employed on a grant from Parkinson's UK, G-0605, awarded to SD and MR and is now a clinical research scientist at Novartis. SD is a senior lecturer in Pharmacology at King's College London, and an expert on neuroprotection in animal models of Parkinson's Disease. MR is a Reader in Pharmacology at the Reading School of Pharmacy, and a neurochemist with a specific interest in glutamate transporters and astrocyte biology.
